# Sex Differences and Long-Term Outcomes in Patients with Left Bundle Branch Area Pacing Compared with Right Ventricular Pacing

**DOI:** 10.3390/jcm14155256

**Published:** 2025-07-24

**Authors:** Po-Wei Yang, Uei Chen, Po-Jui Wu, Shaur-Zheng Chong, Yen-Nan Fang, Yung-Lung Chen, Mien-Cheng Chen, Huang-Chung Chen

**Affiliations:** Division of Cardiology, Department of Internal Medicine, Kaohsiung Chang Gung Memorial Hospital, College of Medicine, Chang Gung University, 123, Dapi Road, Niao-Sung District, Kaohsiung City 833, Taiwan; en780127@cgmh.org.tw (P.-W.Y.); ianchen929@cgmh.org.tw (U.C.); sky1021@cgmh.org.tw (P.-J.W.); shauz@cgmh.org.tw (S.-Z.C.); wideopen@cgmh.org.tw (Y.-N.F.); lung@cgmh.org.tw (Y.-L.C.); chenmien@ms76.hinet.net (M.-C.C.)

**Keywords:** sex, left bundle branch area pacing, clinical outcomes, heart failure hospitalization, mortality

## Abstract

**Background**: Long-term right ventricular pacing (RVP) can cause electrical and mechanical dyssynchrony, resulting in adverse outcomes. Recently, left bundle branch area pacing (LBBAP) has emerged as a physiological pacing modality and is considered a promising alternative. To date, the long-term outcomes of LBBAP compared with RVP, particularly with respect to sex differences, remain unclear. **Methods**: Between January 2017 and July 2024, 1211 patients who underwent de novo pacemaker implantation were enrolled and categorized into RVP (*n* = 789) and LBBAP (*n* = 422). The primary outcome was a composite of all-cause mortality, heart failure hospitalization (HFH), and pacing-induced cardiomyopathy (PICM). Propensity score matching (PSM) was employed to minimize the selection bias and achieve comparability among the study population. A post hoc power analysis based on the observed effect size and sample size showed a power of 80%, confirming sufficient sensitivity to detect group differences. **Results**: After PSM, 764 patients were analyzed. The mean age of the patients was 74.6 ± 10.5 years in RVP and 74.5 ± 9.8 years in LBBAP, respectively, and 52.3% patients were male. Patients with LBBAP had a lower incidence of the primary outcome (8.6% vs. 24.6%, *p* < 0.001), HFH (2.6% vs. 13.6%, *p* < 0.001), and all-cause mortality (6.5% vs. 13.9%, *p* < 0.001) compared with RVP. There were no significant differences in the clinical outcomes, including the primary outcome, HFH and all-cause mortality, between the sexes in the group with either RVP or LBBAP. However, during a 2-year follow-up period for survival analysis, male patients with LBBAP had a significant lower incidence of all the endpoints, whereas female patients with LBBAP had a lower incidence of HFH [HR 0.14 (95% CI 0.06–0.32), *p* = 0.001] compared with those with RVP. **Conclusions**: Regardless of sex, patients with LBBAP had a lower risk of poor clinical outcomes, including HFH and all-cause mortality, compared to those with RVP. Moreover, compared with RVP, LBBAP decreased the risks of all the major endpoints in male patients and the risk of HFH particularly in female patients. Further research is needed to establish the sex-specific responses to LBBAP.

## 1. Introduction

Cardiac pacemakers have been widely used for patients with high-degree atrioventricular conduction block, sick sinus syndrome, or symptomatic bradycardia. Traditionally, right ventricular pacing (RVP) has been the standard pacing modality for many years since 1958 owing to its considerable experience, ease of implantation and procedural safety [1]. However, recent studies have demonstrated that a high pacing burden rate combined with chronic RVP can lead to ventricular dyssynchrony [2,3], resulting in impaired left ventricular contractile function, RV pacing-induced cardiomyopathy (PICM) [4], an increased risk of heart failure hospitalization (HFH), and a higher incidence of atrial arrhythmias, particularly atrial fibrillation [5,6,7]. In recent years, conduction system pacing (CSP), particularly left bundle branch area pacing (LBBAP), has gained increasing attention due to its ability to better align with the physiological electrical conduction pathway. Recent studies revealed that LBBAP has numerous advantages, including improving the synchrony of the left ventricle [8], preserving RV systolic function [9], and lowering the risk of heart failure progression [10,11,12], making it a promising alternative to traditional RVP [13,14]. In addition, LBBAP may reduce the need for future upgrade to biventricular pacing for cardiac resynchronization therapy (CRT) [15,16], and it has even demonstrated superior outcomes compared to biventricular pacing, including lower rates of heart failure hospitalization and greater improvements in the left ventricular ejection fraction [17]. To date, evidence regarding the long-term outcomes of cardiac implantable electronic device (CIED) implantation, particularly in relation to mortality and complication rates, across different sexes and pacing strategies remains inconsistent [18,19,20]. Currently, there is limited long-term evidence evaluating the post-pacemaker implantation outcomes in heart failure and the incidence of PICM with respect to sex differences in terms of the two pacing strategies. Therefore, this study aimed to investigate the long-term clinical outcomes among different sexes and pacing modalities.

## 2. Material and Methods

### 2.1. Study Population

**Figure 1:**
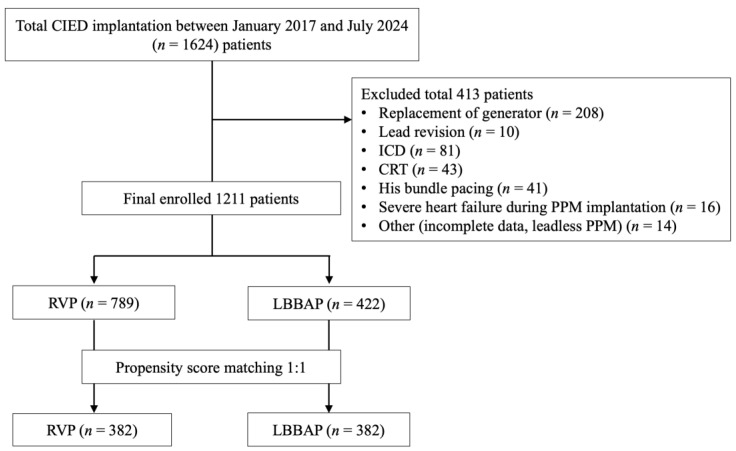
Flowchart of patient enrollment in this study. Abbreviations: CIED, cardiac implantable electronic device; CRT, cardiac resynchronization therapy; ICD, implantable cardioverter defibrillator; LBBAP, left bundle branch area pacing; PPM, permanent pacemaker; RVP, right ventricular pacing.

This retrospective cohort study recruited 1624 consecutive patients receiving CIEDs from our institute between January 2017 and July 2024. The study included patients who underwent de novo pacemaker implantation with either RVP or LBBAP, as recorded in the clinical archives of the Department of Cardiology at Kaohsiung Chang Gung Memorial Hospital. After excluding 413 patients, consisting of 208 patients with replacement of the generator, 81 patients with implantable cardioverter defibrillators (ICDs), 43 patients with CRT, 16 patients with recurrent episodes of HFH prior to pacemaker implantation or with critical conditions such as uncontrolled infection, severe hemodynamic shock, requirement for mechanical circulatory support, or ongoing inotropic agent use at the time of the index procedure, and 65 patients with other issues, 1211 patients were enrolled and were divided into two groups by different pacing modalities: patients with RVP (*n* = 789) and patients with LBBPA (*n* = 422) (Figure 1). Demographic and clinical data, including sex, age, comorbidities, and indications for pacemaker implantation, were collected. The comorbidities considered for analysis included coronary artery disease (CAD), heart failure, diabetes mellitus, hypertension, hyperlipidemia, peripheral artery disease, valvular heart disease (VHD), stroke/transient ischemic attack (TIA), previous coronary artery bypass grafting (CABG), atrial fibrillation, chronic kidney disease, malignancy, chronic obstructive pulmonary disease (COPD) and asthma. This study was conducted in accordance with the principles outlined in the Declaration of Helsinki. This study was approved by the institution review board (approval number: 202500321B0) and ethical approval was obtained from the Ethical Committee of Kaohsiung Chang Gung Memorial Hospital.

### 2.2. Procedural Techniques for Left Bundle Branch Area Pacing

Twelve-lead surface electrocardiograms and intracardiac electrograms were simultaneously recorded using a multichannel Prucka CardioLab system (GE Medical Systems Information Technologies, Milwaukee, WI, USA). Prior to the left bundle branch pacing (LBBP) procedure, the ventricular septal thickness was evaluated via echocardiography. The delivery sheath (C315 His, Medtronic Inc., Minneapolis, MN, USA) was positioned approximately 1.0 to 1.5 cm distal to the His bundle region or the septal leaflet of the tricuspid valve, oriented toward the right ventricular apex, under fluoroscopic guidance in the right anterior oblique 30° view. A 4.1-French lumenless pacing lead (SelectSure 3830, Medtronic Inc., Minneapolis, MN, USA) was introduced through the C315 His sheath, with the lead tip positioned against the interventricular septum. Initial pacing at 5.0 V with a pulse width of 0.4 ms was performed to elicit a characteristic “W”-shaped QRS morphology in lead V1, with the notch positioned near the nadir, indicative of septal engagement. The lead was then actively fixed by perpendicular advancement into the left ventricular septum, and the fixation was halted upon confirmation of left bundle branch (LBB) capture [21]. As previously described, the following criteria were used to confirm direct LBB capture [21]: (1) a paced QRS morphology consistent with a right bundle branch block (RBBB) pattern; (2) the presence of an LBB potential; (3) a pacing stimulus-to-left ventricular activation time (S-LVAT) that either shortens abruptly with increased output or remains minimal and constant at both low and high outputs; and (4) demonstration of both selective and non-selective LBBP. To differentiate LBBP from left ventricular septal pacing (LVSP), LBBP was defined as either [22] (1) the presence of an LBB potential and S-LVAT ≤ 85 ms, or (2) the absence of an LBB potential with S-LVAT ≤ 70 ms. In contrast, LVSP was defined as either (1) the presence of an LBB potential with S-LVAT > 85 ms, or (2) the absence of an LBB potential with S-LVAT > 70 ms. LBBAP was comprised of LBBP and LVSP.

### 2.3. Study Endpoint

This study assessed several clinical outcomes, including hospitalization due to heart failure or acute coronary syndrome (ACS), PICM, stroke/TIA, cardiovascular mortality, and all-cause mortality. Cardiovascular events included HFH and ACS hospitalization. The primary outcome comprised PICM, HFH, and all-cause mortality. The secondary outcomes were hospitalization due to ACS, stroke/TIA, and cardiovascular mortality.

### 2.4. Statistical Analyses

Comparisons between groups, including sex-based differences, were performed using the chi-square or Fisher’s exact test for categorical variables and the t-test for continuous variables. Propensity score matching (PSM) analysis was employed to ensure the comparability of the baseline characteristics between the observed patient groups, utilizing a 1:1 matching approach with the greedy method. The standardized mean difference (SMD) was calculated to assess the balance of covariates following propensity score matching and an SMD less than 0.1 indicated balance. Kaplan–Meier survival analysis was conducted to evaluate the patient outcomes over one-year and two-year follow-up periods, commencing from the initial pacemaker implantation, with statistical comparisons performed using the Log-rank test. The risks of time-to-event outcomes between groups were evaluated using the Cox proportional hazards model. To identify independent predictors of clinical outcomes, we conducted a forward stepwise Cox regression analysis, which allowed for the sequential inclusion of variables based on the statistical contribution to the model. A two-sided *p*-value of less than 0.05 was considered statistically significant.

To evaluate the adequacy of our sample size and the statistical power required to detect differences in the primary and secondary outcomes, we performed a post hoc power analysis using the PASS software (NCSS, LLC). Parameters required for the analysis, including the observed number of events in each subgroup, two-tailed test with an alpha level of 0.05, duration of follow-up, hazard ratios and survival proportions of all events, were entered into the software. Depending on data availability, we employed different methods, either the Log-rank test based on the hazard ratios or the Log-rank test based on the survival proportions, for the calculation. All the computed power values exceeded 0.80, indicating that this study had sufficient power to detect clinically relevant differences across study groups and the differences were unlikely to be due to type II error. All the statistical analyses were conducted using SPSS (version 26), MedCalc (version 23.1.1; MedCalc Software Ltd., Ostend, Belgium), NCSS Statistical Software (version 12), PASS Software (version 14.0.7) and R [version 3.6.1 (R Core Team, 2020)].

## 3. Results

### 3.1. Baseline Characteristics of Total Cohort with RVP and LBBAP

Table 1 and Table S1 list the clinical characteristics of the study population. Before PSM, the mean age of the patients was 74.9 ± 10.8 years in RVP and 74.6 ± 9.7 years in LBBAP, and nearly 50.3% of the study patients were male. Hypertension was the most common comorbidity in both sexes, with a prevalence of 74.7% in males and 75.6% in females. Several other comorbid conditions, including CAD, cerebrovascular accident, malignancy, and chronic obstructive pulmonary disease (COPD)/asthma, were more frequently observed in male patients. In contrast, peripheral artery disease (PAD) appeared to be slightly more prevalent among female patients. Regarding the indications for pacemaker implantation, complete atrioventricular (AV) block was more commonly observed in male patients (50.7% vs. 41.7% in females), whereas sick sinus syndrome was a more frequent indication among female patients (57% vs. 48.3% in males). With regard to pharmacological treatment for heart failure and coronary artery disease, diuretic agents were more commonly prescribed in female patients compared to males (31.2% vs. 25.9%, respectively). In contrast, sodium–glucose co-transporter 2 inhibitors (SGLT2i) were used more frequently among male patients (12.8% vs. 8.3% in females), primarily for the management of diabetes mellitus. After performing 1:1 PSM, the baseline characteristics between the RVP and LBBAP groups were well-balanced, as shown in Table 1, with all the SMDs being less than 0.1—indicating an adequate covariate balance. Following matching, a total of 382 matched pairs of patients were included in the final analysis.

### 3.2. Clinical Outcomes of the Matched Cohort After PSM

**Figure 2:**
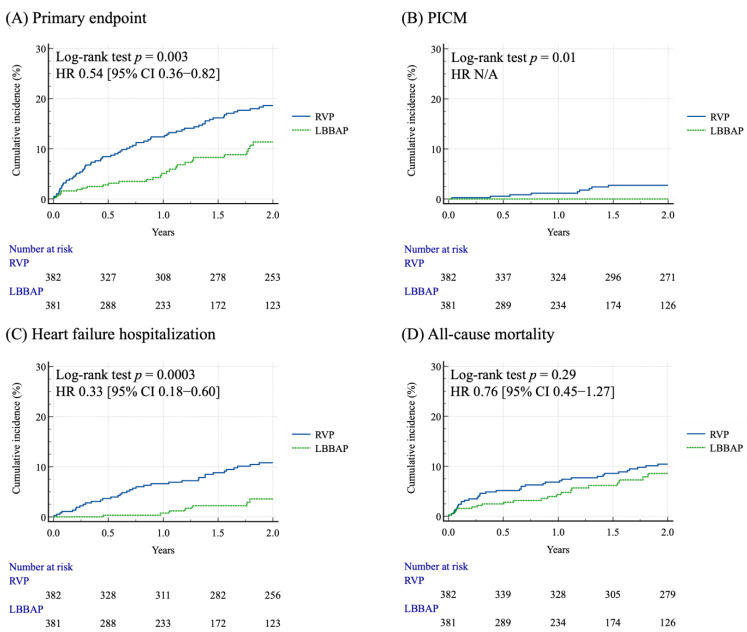
The Kaplan–Meier event-free survival curves of the primary endpoint (**A**), PICM (**B**), heart failure hospitalization (**C**) and all-cause mortality (**D**) between the RVP and LBBAP groups after propensity score matching during the 2-year follow-up period.

During the 5-year follow-up period, the incidence of the primary outcome was lower in patients with LBBAP compared to patients with RVP (8.6% vs. 24.6%, *p* < 0.001) (Table 2). The incidences of PICM, HFH, and all-cause mortality were still lower in patients with LBBAP compared to patients with RVP (0% vs. 3.7%, *p* < 0.001; 2.6% vs. 13.6%, *p* < 0.001; 6.5% vs. 13.9%, *p* < 0.001, respectively) (Table 2). The incidences of PICM, cardiovascular events, stroke/TIA, cardiovascular mortality, and all-cause mortality did not differ between male and female patients in either the RVP or LBBAP group (Table 3). The Kaplan–Meier curve analysis for the clinical outcomes over the two-year follow-up period after PSM for the two groups are shown in Figure 2. Patients with LBBAP had a lower cumulative incidence of primary composite endpoint, PICM, and HFH compared to patients with RVP (*p* = 0.003, *p* = 0.01, and *p* = 0.0003, respectively) (Figure 2A–C). The cumulative incidence of all-cause mortality did not differ between patients with LBBAP and RVP (*p* = 0.29) (Figure 2D).

### 3.3. Sex-Based Survival Analysis in Matched Cohort

**Figure 3:**
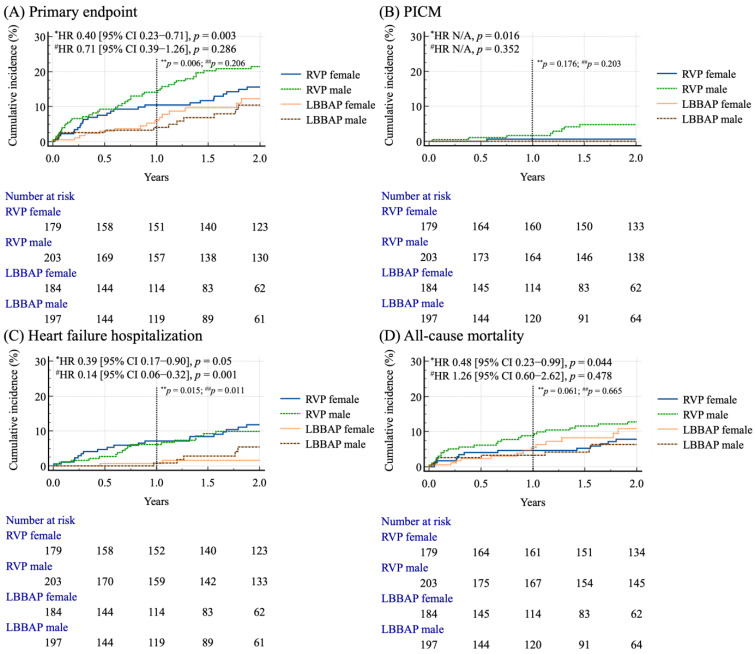
Cumulative incidence rates of the primary endpoint (**A**), PICM (**B**), heart failure hospitalization (**C**) and all-cause mortality (**D**) across the different stimulation strategies and genders after propensity score matching during the 2-year follow-up period. * Male (LBBAP vs. RVP); ** Male (LBBAP vs. RVP) in the 1-year follow-up period; ^#^ Female (LBBAP vs. RVP); ^##^ Female (LBBAP vs. RVP) in the 1-year follow-up period.

The Kaplan–Meier curve analysis based on the sex differences for clinical outcomes for the two groups is shown in Figure 3 and Figure S1. Only male patients with LBBAP had a significantly lower cumulative incidence of the primary composite endpoint in both the 1-year and 2-year follow-up periods (Log-rank test, *p* = 0.006; *p* = 0.003, respectively) (Figure 3A), and PICM in the 2-year follow-up period (Log-rank test, *p* = 0.016) (Figure 3B), compared to male patients with RVP. Both male and female patients with LBBAP had a lower cumulative incidence of HFH compared with the RVP group in the 1-year follow-up period (Log-rank test, *p* = 0.015; *p* = 0.011, respectively) and 2-year follow-up period (Log-rank test, *p* = 0.05; *p* = 0.001, respectively) (Figure 3C). During the 1-year follow-up period, there was no significant difference in the cumulative incidence of all-cause mortality between male and female patients, regardless of the pacing modality (LBBAP or RVP). However, at the 2-year follow-up, male patients receiving LBBAP exhibited a significant 52% reduction in all-cause mortality compared to their counterparts [HR 0.48 (95% CI, 0.23–0.99), *p* = 0.044] (Figure 3D).

### 3.4. Identification of Predictors of Primary Composite Endpoint and Heart Failure Hospitalization

After matching, the clinical variables that were statistical significantly associated with the primary composite endpoint were age, hypertension, diabetes mellitus, CAD, HF, valvular heart disease, atrial fibrillation, chronic kidney disease, end-stage renal disease, peripheral artery disease, and LBBAP in the univariate Cox regression analysis (Table 4). In the multivariate Cox regression analysis, age [HR 1.04 (95% CI 1.01–1.06), *p* < 0.001], CAD [HR 2.24 (95% CI 1.49–3.37), *p* < 0.001], HF [HR 1.64 (95% CI, 1.002–2.59), *p* = 0.049], valvular heart disease [HR 2.14 (95% CI, 1.39–3.29), *p* = 0.001], atrial fibrillation [HR 1.51 (95% CI, 1.05–2.17), *p* = 0.024], chronic kidney disease [HR 1.96 (95% CI, 1.30–2.95), *p* = 0.001], end-stage renal disease [HR 2.22 (95% CI, 1.19–4.12), *p* = 0.012], and peripheral artery disease [HR 2.94 (95% CI, 1.32–6.52), *p* = 0.008] were independent determinants of the primary composite endpoint (Table 4). Notably, LBBAP [HR 0.56 (95% CI 0.37–0.84), *p* = 0.006] was associated with a significantly reduced risk of the primary composite endpoint (Table 4). In the multivariate Cox regression analysis for HFH, hypertension, heart failure, and valvular heart disease were associated with worse outcomes, while LBBAP remained a strong protective factor [HR 0.31 (95% CI 0.15–0.64), *p* = 0.001] (Table S3).

## 4. Discussion

This study provides novel insights into the long-term outcomes of LBBAP compared to RVP, with a particular focus on sex differences. Our main findings indicated that (1) LBBAP was associated with superior clinical outcomes compared to RVP, demonstrating significantly lower risks of the primary composite outcome, including PICM, and HFH (Table 2 for 5-year event rate and Figure 2 for 2-year survival rate); (2) LBBAP consistently acted as a protective factor to reduce by 44% the risk of the primary composite endpoint (Table 4) and by 69% the risk of heart failure hospitalization (Table S3); and (3) during the 1-year follow-up period, males with LBBAP had a lower incidence of both the primary composite outcome and HFH, whereas females with LBBAP had a lower incidence of HFH only. However, after the 2-year follow-up, LBBAP offered distinct advantages with regard to the sex differences, in which male patients experienced lower risks of all the endpoints, including the primary composite outcome, PICM, HFH and all-cause mortality, while female patients had a reduced incidence of HFH compared to those with RVP (Figure 3 and Figure S1).

### 4.1. Sex-Based Demographic Disparities

Previous studies have shown that women receiving pacemakers were generally older than men [23,24,25], a trend also observed in our cohort (age of males 74.2 ± 10.0 years, age of females 75.3 ± 10.8 years; *p* = 0.072, Table S1). This age difference may be attributed to women’s longer life expectancy and lower burden of comorbidities, particularly cardiovascular diseases. Consistent with prior research [24,25,26], our study found men had a higher prevalence of coronary artery disease than women (26.6% vs. 16.3%, *p* < 0.001, Table S1), possibly related to the cardioprotective effects of estrogen. Estrogen helps maintain the calcium balance, inhibits mitochondrial apoptosis, and reduces arrhythmic events by modulating cardiac ion channels, especially after ischemia–reperfusion injury [27,28,29,30]. Additionally, women may undergo device implantation less frequently due to greater procedural anxiety and a higher likelihood of declining therapy [31,32], potentially contributing to their older age at implantation.

### 4.2. Sex-Based and Pacing-Modality-Related Outcomes

This study highlights the clinical advantages of LBBAP over RVP, particularly in reducing HFH, PICM, and the primary composite endpoint. Conventional RVP can cause interventricular and intraventricular dyssynchrony, leading to impaired ventricular function and increased risks of heart failure and mortality [33,34]. In contrast, LBBAP promotes more physiological ventricular activation by minimizing electrical dyssynchrony, resulting in fewer HFH events [8,11,13]. A multicenter prospective study found that LBBAP significantly lowered the incidence of heart failure events in patients with atrioventricular block (2.6% vs. 10.8%, *p* < 0.001) [16]. Another large cohort study comparing conduction system pacing (CSP) to dual-chamber RVP showed that CSP, including LBBAP and His bundle pacing, was associated with lower HFH (HR 0.70, *p* = 0.02) and six-month mortality (HR 0.66, *p* < 0.0001) [11]. Furthermore, LBBAP has been proposed to reduce the need for future upgrade to biventricular pacing for CRT in patients with heart failure [15,16]. Additionally, prior studies have reported that LBBAP may provide more favorable pacing and echocardiographic parameters, such as a narrower QRS duration, shorter paced left ventricular activation time (LVAT) [15], greater improvement in LVEF [17], higher ventricular R-wave amplitudes, and pacing thresholds [12,35] comparable to those observed with RVP or biventricular pacing. These benefits may be attributed to LBBAP leading to more preserved right and left ventricular systolic function and myocardial work [8,9]. Notably, LBBAP was associated with a reduction in HFH in both male and female patients at the 1-year follow-up, with this beneficial effect persisting through the 2-year period, as shown in Figure 3 and Figure S1. These findings are consistent with the previously published literature reporting similar outcomes.

### 4.3. Sex-Based and Pacing-Modality-Related Survival Analysis

After sex stratification, no significant differences in cardiovascular events, ACS or HF hospitalizations, cerebral vascular accident, PICM, or all-cause mortality were observed between males and females within either the RVP or LBBAP group (Table 3), although the overall event rates were consistently lower in the LBBAP cohort. Our findings are consistent with a prior study, which reported no sex-related differences in device-related complications, mortality, stroke, or one-year survival in an Australian registry of 5360 CIED recipients [25]. However, LBBAP showed sex-specific benefits in our cohort: only male patients who received LBBAP exhibited a lower incidence of the primary composite outcome and HFH compared to those with RVP during the 1-year follow-up period, as illustrated in Figure 3 and Figure S1. With extended follow-up to 2 years, the sex-specific differences in the clinical benefit became more pronounced. Male patients undergoing LBBAP exhibited significantly reduced risks of the primary composite outcome, PICM, HFH, and all-cause mortality. In contrast, among female patients, the benefit of LBBAP was limited to a significant reduction in HFH.

Revisiting this issue, the observed sex-based differences in the clinical outcomes align with findings from previous studies, which revealed that men with LBBAP had a lower risk of HFH and all-cause mortality compared to men with biventricular pacing, whereas there was no difference between the two strategies in women [36]. While earlier reviews found no sex-based mortality differences post-CIED implantation [25,36], our study observed a non-significant trend of a higher mortality rate in women with LBBAP (8.1% vs. 5.1%, *p* = 0.231, Table 3), largely driven by non-procedural causes such as infection and sepsis (5.4% vs. 2.0%, Table 3). Previous studies suggested women may be more vulnerable to post-implant infections due to immune and hormonal differences [18,19], as well as anatomical factors like smaller cardiac structures, which may complicate procedures [20]. Despite the higher perioperative morbidity in women, long-term mortality is often higher in men [37]. A large Australian study also found higher 30-day mortality in men after implantation; however, this sex-related difference disappeared after adjusting for underlying diagnoses and the specific pacing group, suggesting the potential influence of selection bias [23]. These mixed findings underscore the need for further research to clarify the biological, procedural, and socio-demographic factors influencing sex-based outcomes after CIED implantation.

### 4.4. Potential Influencing Factors for HFH, PICM, and All-Cause Mortality in the Matched Cohort

In our matched cohort analysis, the univariate analysis identified advanced age and comorbidities, such as hypertension, diabetes, CAD, heart failure, VHD, CKD, and PAD, as significant risk factors for HFH, PICM, and mortality (Table 4). In the multivariate models, heart failure and VHD emerged as the strongest independent predictors of adverse outcomes. In contrast, LBBAP consistently showed a protective effect against heart failure and the primary composite outcomes, while sex was not associated with poor outcomes. After PSM of this cohort, including sex, no significant difference in survival between males and females was observed. This contrasts with a prior single-center study where being female was associated with better survival post-pacemaker implantation, which lacked propensity score matching and included more cardiovascular comorbidities in males [26].

In the present study, VHD and heart failure emerged as the strongest independent predictors of adverse clinical outcomes. It is well-established that traditional RV pacing alters the physiological pattern of ventricular activation, causing electrical and mechanical dyssynchrony. This dyssynchronous activation may lead to impaired contractility, elevated filling pressures, and cardiac remodeling, and it may also exacerbate pre-existing valvular regurgitation, further contributing to heart failure progression [38,39]. In one study of 125 AVB patients, RVP increased the prevalence of moderate-to-severe tricuspid regurgitation from 8.7% at baseline to 31.6% after implantation (*p* < 0.001); the prevalence of mild and moderate mitral regurgitation also increased from 29.6% at baseline to 54% after the procedure (*p* < 0.001) [40]. However, no sex-based differences in valvular regurgitation were observed in this cohort [40]. Our study did not assess the sex-specific effects in patients with VHD due to limited echocardiographic data during the follow-up period. Given prior evidence of structural and functional sex differences in the valvular anatomy and LV geometry, further large-scale studies are needed to clarify the sex-based effects in this subgroup.

### 4.5. Study Limitations

Despite its strengths, our study has several limitations that should be acknowledged. First, electrocardiographic parameters and echocardiographic measurements, including the QRS duration and the severity of valvular regurgitation, were not regularly collected in this study. Second, the retrospective and single-center design of this study inherently introduces the possibility of selection bias, information bias, and unmeasured confounding factors. For this reason, PSM analysis was utilized to balance the bias between the observed patient groups. Third, our study population consisted exclusively of Asian patients, which may limit the generalizability of our findings to other ethnic or regional populations, given the known differences in cardiovascular risk profiles and device implantation practices across populations. Fourth, the power to detect differences in all-cause mortality in Figure 2 was slightly below the target level (estimated power = 0.7854), with the minimum required sample size exceeding 793 cases. Similar limitations were identified in the subgroup analysis after stratification by sex, particularly in the comparison between female patients receiving RVP and those receiving LBBAP (Figure 3). In these subgroups, the estimated power required to detect differences in the primary composite outcome, PICM, and all-cause mortality did not reach the desired threshold. This limitation may partly explain the absence of statistically significant differences in these comparisons, despite the numerically favorable trends. Therefore, these results should be interpreted with caution, and future investigations with larger, adequately powered, sex-stratified cohorts will be essential to confirm and expand upon these observations.

## 5. Conclusions

This study demonstrated that LBBAP was associated with significantly improved long-term clinical outcomes compared to RVP, including lower incidences of PICM, HFH, and all-cause mortality. LBBAP consistently emerged as an independent protective factor against adverse clinical outcomes. About the sex-based differences, LBBAP conferred greater clinical benefit in male patients, who demonstrated reduced risks across all the major endpoints after two years of follow-up, whereas in female patients, the benefit was limited to a reduction in HFH. Further large-scale, multicenter prospective studies are warranted to confirm these observations and guide individualized pacing therapy.

## Figures and Tables

**Table 1:** Baseline characteristics of patients with RVP and LBBAP before and after propensity score matching.

	Before PSM (*n* = 1211)			After PSM (*n* = 764)		
Variables	RVP (*n* = 789)	LBBAP (*n* = 422)	SMD	RVP (*n* = 382)	LBBAP (*n* = 382)	SMD
Male	392 (49.7)	217 (51.4)	0.035	203 (53.1)	197 (51.6)	0.031
Age, years	74.9 (10.8)	74.6 (9.7)	0.024	74.6 (10.5)	74.5 (9.8)	0.002
BMI, kg/m^2^	24.8 (3.9)	25.12 (4.0)	0.069	25.0 (3.9)	25.1 (3.9)	0.024
Hypertension	595 (75.4)	315 (74.6)	0.018	283 (74.1)	283 (74.1)	<0.001
Diabetes	332 (42.1)	166 (39.3)	0.056	138 (36.1)	142 (37.2)	0.022
Dyslipidemia	312 (39.5)	175 (41.5)	0.039	155 (40.6)	153 (40.1)	0.011
CAD	176 (22.3)	84 (19.9)	0.059	74 (19.4)	77 (20.2)	0.020
HF history	126 (16.0)	55 (13.0)	0.083	45 (11.8)	45 (11.8)	<0.001
VHD ^a^	133 (16.9)	79 (18.7)	0.049	66 (17.3)	71 (18.6)	0.034
AF	296 (37.5)	150 (35.5)	0.041	143 (37.4)	136 (35.6)	0.038
CVA	142 (18.0)	81 (19.2)	0.031	74 (19.4)	74 (19.4)	<0.001
CKD ^b^	244 (30.9)	79 (18.7)	0.285	73 (19.1)	75 (19.6)	0.013
ESRD ^c^	78 (9.9)	27 (6.4)	0.128	23 (6.0)	27 (7.1)	0.042
PAD	22 (2.8)	5 (1.2)	0.115	9 (2.4)	5 (1.3)	0.078
Malignancy	161 (20.4)	72 (17.1)	0.086	68 (17.8)	68 (17.8)	<0.001
CV op history	59 (7.5)	33 (7.8)	0.013	26 (6.8)	29 (7.6)	0.030
COPD/asthma	98 (12.4)	34 (8.1)	0.144	30 (7.9)	33 (8.6)	0.029
Beta-blocker	337 (42.7)	207 (49.1)	0.127	174 (45.5)	183 (47.9)	0.047
RAS blockade	469 (59.4)	261 (61.8)	0.049	231 (60.5)	232 (60.7)	0.005
Diuretic	268 (34.0)	78 (18.5)	0.358	76 (19.9)	77 (20.2)	0.007
Statin	349 (44.2)	216 (51.2)	0.140	187 (49.0)	186 (48.7)	0.005
SGLT2i	57 (7.2)	71 (16.8)	0.298	43 (11.3)	43 (11.3)	<0.001
OADs	248 (31.4)	147 (34.8)	0.072	118 (30.9)	122 (31.9)	0.023
Insulin	53 (6.7)	24 (5.7)	0.043	23 (6.0)	19 (5.0)	0.046

Data are presented as the mean ± SD or number (%) of patients. ^a^ Defined as moderate, severe regurgitation or stenosis of aortic, mitral or tricuspid valves. ^b^ Defined as eGFR lower than 60 mL/min/1.73 m^2^ without renal replacement therapy. ^c^ Defined as the need for peritoneal dialysis, hemodialysis, or renal transplantation. Abbreviations: AF, atrial fibrillation; BMI, body mass index; CAD, coronary artery disease; CV, cardiovascular; CVA, cerebral vascular accident; CKD, chronic kidney disease; COPD, chronic obstruction pulmonary disease; ESRD, end-stage renal disease; HF, heart failure; OADs, oral antidiabetic agent; PSM, propensity score matching; PAD, peripheral artery disease; RAS, renin–angiotensin system; SMD, standard mean difference; SD, standard deviation; SGLT2i, sodium–glucose transport protein 2 inhibitor; VHD, valvular heart disease.

**Table 2:** Clinical outcomes of patients with RVP and LBBAP after PSM during the 5-year follow-up period.

	Stratified by Different Pacing Strategy		
	RVP (*n* = 382)	LBBAP (*n* = 382)	*p* Value
Cardiovascular events	70 (18.3)	20 (5.2)	<0.001
HF hospitalization	52 (13.6)	10 (2.6)	<0.001
ACS hospitalization	25 (6.5)	11 (2.9)	0.017
CVA *	13 (3.4)	9 (2.4)	0.387
PICM	14 (3.7)	0 (0)	<0.001
Primary outcomes	94 (24.6)	33 (8.6)	<0.001
Cardiovascular mortality	9 (2.4)	2 (0.5)	0.034
All-cause mortality	53 (13.9)	25 (6.5)	<0.001

Data are presented as the number (%) of patients. * CVA including stroke/transient ischemic attack (TIA). Abbreviations: ACS, acute coronary syndrome; CVA, cerebral vascular accident; HF, heart failure; PICM, pacing-induced cardiomyopathy; PSM, propensity score matching.

**Table 3:** Events following pacemaker implantation in the RVP and LBBAP cohorts stratified by sex after PSM during the 5-year follow-up period.

	Stratified by Different Pacing Modalities and Gender					
	RVP (*n* = 382)			LBBAP (*n* = 382)		
	Male (*n* = 203)	Female (*n* = 179)	*p* Value	Male (*n* = 197)	Female (*n* = 185)	*p* Value
Cardiovascular events	36 (17.7)	34 (19.0)	0.751	10 (5.1)	10 (5.4)	0.885
HF hospitalization	24 (11.8)	28 (15.6)	0.277	6 (3.0)	4 (2.2)	0.587
ACS hospitalization	13 (6.4)	12 (6.7)	0.906	5 (2.5)	6 (3.2)	0.68
CVA	10 (4.9)	3 (1.7)	0.08	7 (3.6)	2 (1.1)	0.101
PICM	9 (4.4)	5 (2.8)	0.395	0 (0)	0 (0)	N/A
Primary outcomes	53 (26.1)	41 (22.9)	0.468	14 (7.1)	19 (10.3)	0.271
Cardiovascular mortality	3 (1.5)	6 (3.4)	0.228	2 (1.0)	0 (0)	0.103
All-cause mortality	32 (15.8)	21 (11.7)	0.255	10 * (5.1)	15 ^^^ (8.1)	0.231

Data are presented as the number (%) of patients. * Sepsis, infection in LBBAP male: 4 (2%). ^ Sepsis, infection in LBBAP female: 10 (5.4%). Abbreviations: ACS, acute coronary syndrome; CVA, cerebral vascular accident; HF, heart failure; PICM, pacing-induced cardiomyopathy; PSM, propensity score matching.

**Table 4:** Univariate and multivariate Cox regression analyses of predictors of the primary endpoint in the matched cohort.

	After PSM (*n* = 764)			
Variables	Univariate Analysis		Multivariate Analysis	
	HR (95% CI)	*p* Value	HR (95% CI)	*p* Value
Sex (male)	1.04 (0.73–1.47)	0.814		
Age (years)	1.04 (1.02–1.06)	<0.001	1.04 (1.01–1.06)	<0.001
BMI	0.95 (0.92–1.01)	0.137		
Hypertension	1.84 (1.16–2.92)	0.009	1.06 (0.65–1.73)	0.8
Diabetes mellitus	1.51 (1.06–2.15)	0.02	1.22 (0.84–1.78)	0.287
Dyslipidemia	0.75 (0.52–1.09)	0.134		
Coronary artery disease	2.32 (1.59–3.38)	<0.001	2.24 (1.49–3.37)	<0.001
Heart failure	3.37 (2.22–5.12)	<0.001	1.64 (1.002–2.59)	0.049
Valvular heart disease	3.15 (2.17–4.58)	<0.001	2.14 (1.39–3.29)	0.001
Atrial fibrillation	1.43 (1.009–2.03)	0.044	1.51 (1.05–2.17)	0.024
Cerebral vascular accident	1.29 (0.84–1.98)	0.234		
Chronic kidney disease	2.34 (1.60–3.42)	<0.001	1.96 (1.30–2.95)	0.001
End-stage renal disease	3.28 (1.96–5.50)	<0.001	2.22 (1.19–4.12)	0.012
Peripheral artery disease	6.78 (3.30–13.9)	<0.001	2.94 (1.32–6.52)	0.008
LBBAP	0.58 (0.38–0.88)	0.012	0.56 (0.37–0.84)	0.006

Abbreviation: BMI, body mass index; LBBAP, left bundle branch area pacing; PSM, propensity score matching.

## Data Availability

Datasets are available from the corresponding author upon reasonable request but are not publicly accessible due to ethical restrictions.

## Supplementary Materials

The following supporting information can be downloaded at https://www.mdpi.com/article/10.3390/jcm14155256/s1, Table S1: Baseline demographic profiles of the total cohort; Table S2: Sex- and intervention-specific outcomes assessed in the Kaplan–Meier survival analysis during the 5-year follow up period; Table S3: Univariate and multivariate Cox regression analyses of predictors of heart failure hospitalization in the matched cohort; Figure S1: Cumulative incidence rates of the primary endpoint (Panel A, male; Panel B, female) and heart failure hospitalization (Panel C, male; Panel D, female) across different stimulation strategies and genders after propensity score matching during the 5-year follow-up period. * Analyzed in a 1-year follow-up period.

## Abbreviations

The following abbreviations are used in this manuscript:

ACS	Acute coronary syndrome
CRT	Cardiac resynchronization therapy
CIED	Cardiac implantable electronic devices
HFH	Heart failure hospitalization
LBBAP	Left bundle branch area pacing
PICM	Pacing-induced cardiomyopathy
RVP	Right ventricular pacing
