# Supplementary material for: Sex Differences and Long-Term Outcomes in Patients with Left Bundle Branch Area Pacing Compared with Right Ventricular Pacing
Source: J Clin Med. 2025 Jul 24;14(15):5256. doi: 10.3390/jcm14155256 (PMC12347622; doi:10.3390/jcm14155256)
Supplement: Supplementary file 1 [file jcm-14-05256-s001.zip › jcm-3674037-supplementary.pdf]

Table S1. Baseline demographic profiles for total cohort.

Parameter	Stratified by sex (n=1211)		
	Male (n = 609; 50.3%)	Female (n = 602; 49.7%)	P value
Age, years	74.2 (10.0)	75.3 (10.8)	0.072
BMI, kg/m^2	25.2 (3.65)	24.6 (4.20)	0.01
Referral source			
Emergency department	376 (61.7)	223 (37)	0.662
Outpatient department	233 (38.3)	379 (63)	
Indication for PPM			
Sick sinus syndrome	294 (48.3)	343 (57)	
Complete AV block	309 (50.7)	251 (41.7)	**0.007**
Others[a]	6 (1.0)	8 (1.3)	
Ventricular pacing burden			
<20%	140 (23.0)	188 (31.2)	
≥20%	230 (37.8)	166 (27.6)	**<0.001**
No data	239 (39.2)	248 (41.2)	
Comorbidities			
Hypertension	455 (74.7)	455 (75.6)	0.727
Diabetes mellitus	266 (43.7)	232 (38.5)	0.069
Dyslipidemia	242 (39.7)	245 (40.7)	0.733
CAD	162 (26.6)	98 (16.3)	**<0.001**
HF history	94 (15.4)	87 (14.5)	0.631
EF≥50%	51 (8.4)	64 (10.6)	
41%<EF<49%	16 (2.6)	11 (1.8)	
EF≤40%	27 (4.4)	12 (2.0)	
VHD[1]	101 (16.6)	111 (18.4)	0.396
AF	236 (38.8)	210 (34.9)	0.163
CVA	126 (20.7)	97 (16.1)	**0.04**
CKD[2]	165 (27.1)	158 (26.2)	0.739
ESRD[3]	54 (8.9)	51 (8.5)	0.807
PAD	7 (1.1)	20 (3.3)	**0.01**
Malignancy	133 (21.8)	100 (16.6)	**0.021**
CV op history[b]	46 (7.6)	46 (7.6)	0.954
COPD/Asthma	84 (13.8)	48 (8.0)	**0.001**
HF/CAD medication			
Beta-blocker	262 (43.0)	282 (46.8)	0.181
RAS blockade[c]	359 (58.9)	371 (61.6)	0.341
Diuretic	158 (25.9)	188 (31.2)	**0.042**
Statin	297 (48.8)	268 (44.5)	0.138
SGLT2i	78 (12.8)	50 (8.3)	**0.011**
For DM	43 (7.1)	27 (4.5)	
For HF	30 (4.9)	19 (3.2)	
For CKD	5 (0.8)	4 (0.7)	
DM medication			
OADs[d]	212 (34.8)	183 (30.4)	0.101
Insulin	40 (6.6)	37 (6.1)	0.764

Data are presented as mean ± SD or number (%) of patients

[1]Defined as moderate, severe regurgitation or stenosis of aortic, mitral or tricuspid valves

[2]Defined as eGFR lower than 60 mL/min/1.73m^2 without renal replacement therapy

[3]Defined as the need for peritoneal dialysis, hemodialysis, or renal transplantation

[a]Others PPM indication: including symptomatic sinus bradycardia, vasovagal response with asystole (cardio-inhibition type)

[b]CV op: including CABG, TAVI, any valve operation

[c]RAS blockade: including ACEi/ARB/ARNi

[d]OADs: including biguanide, DPP4i, SU, TZD (without SGLT2)

Abbreviation: AF, atrial fibrillation; BMI, body mass index; CAD, coronary artery disease; CVA, cerebral vascular accident; CKD, chronic kidney disease; COPD, chronic obstruction pulmonary disease; DM, diabetes mellitus; ESRD, end stage renal disease; EF, ejection fraction; HF, heart failure; OADs, oral antidiabetic agent; PPM, permanent pacemaker; PSM, propensity score matching; PAD, peripheral artery disease; RAS, renin-angiotensin system; SGLT2i, sodium-glucose transport protein 2 inhibitor; VHD, valvular heart disease

Table S2. Sex and intervention-specific outcomes assessed by Kaplan-Meier survival analysis during the 5-year follow up period.

	Male (LBBAP vs RVP#)		Female (LBBAP vs RVP#)		RVP (male vs female*)		LBBAP (male vs female*)	
	HR (95% CI)	P value	HR (95% CI)	P value	HR (95% CI)	P value	HR (95% CI)	P value
Primary outcomes	0.47 (0.28-0.78)	0.004	0.79 (0.47-1.33)	0.471	1.18 (0.76-1.83)	0.409	0.70 (0.39-1.26)	0.334
PICM	N/A	0.014	N/A	0.188	1.73 (0.47-6.26)	0.32	N/A	N/A
HF hospitalization	0.49 (0.23-1.03)	0.104	0.27 (0.12-0.57)	0.005	0.78 (0.42-1.44)	0.385	1.44 (0.61-3.39)	0.56
All-cause mortality	0.57 (0.30-1.09)	0.064	1.26 (0.64-2.45)	0.321	1.40 (0.80-2.43)	0.228	0.63 (0.30-1.33)	0.264

#RVP; *Female as control group

Abbreviation: HF, heart failure; PICM, pacing induced cardiomyopathy

Table S3. Univariate and multivariate Cox regression analysis of predictors of heart failure hospitalization in the matched cohort.

Variables	After PSM (N = 764)			
	Univariate analysis		Multivariate analysis	
	HR (95% CI)	P value	HR (95% CI)	P value
Sex (male)	0.87 (0.53-1.43)	0.589		
Age (years)	1.03 (1.009-1.06)	**0.01**	1.01 (0.98-1.04)	0.4
BMI	0.96 (0.90-1.03)	0.347		
Hypertension	2.67 (1.27-5.63)	**0.009**	2.30 (1.05-5.05)	**0.037**
Diabetes mellitus	0.85 (0.49-1.45)	0.556		
Dyslipidemia	0.70 (0.41-1.20)	0.197		
Coronary artery disease	1.66 (0.92-2.97)	0.08		
Heart failure	4.63 (2.63-8.15)	**<0.001**	2.16 (1.15-4.05)	**0.016**
Valvular heart disease	6.28 (3.77-10.4)	**<0.001**	4.61 (2.59-8.22)	**<0.001**
Atrial fibrillation	1.41 (0.85-2.34)	0.173		
Cerebral vascular accident	1.06 (0.55-2.03)	0.86		
Chronic kidney disease	2.10 (1.20-3.68)	**0.009**	1.59 (0.87-2.92)	0.13
End stage renal disease	2.82 (1.28-6.24)	**0.01**	1.23 (0.45-3.35)	0.683
Peripheral artery disease	7.69 (2.78-21.2)	**<0.001**	3.00 (0.87-10.2)	0.08
LBBAP	0.34 (0.17-0.68)	**0.002**	0.31 (0.15-0.64)	**0.001**

Abbreviation: BMI, body mass index; LBBAP, left bundle branch area pacing; PSM, propensity score matching

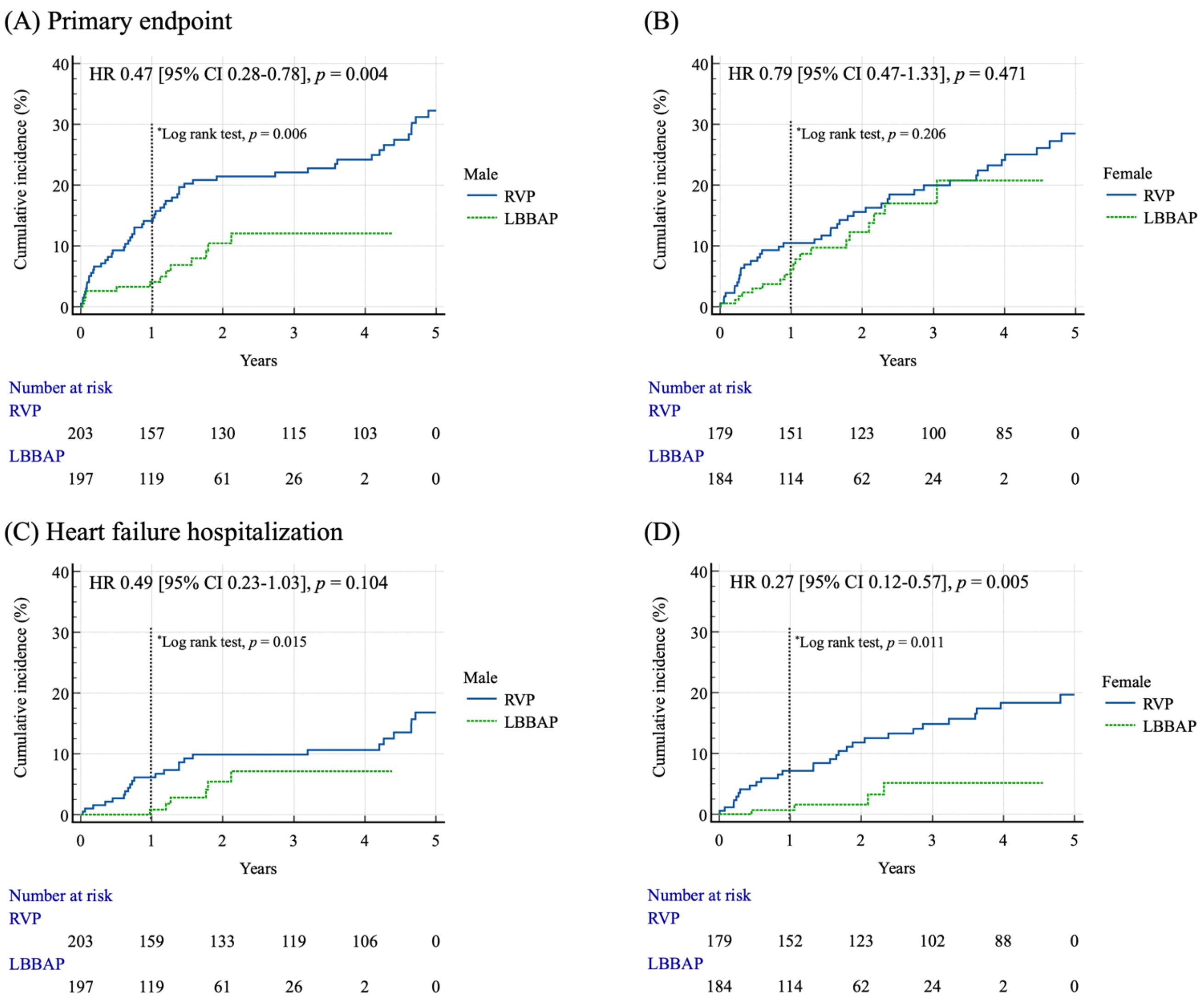

Figure S1. Cumulative incidence rates of the primary endpoint (Panel A, male; Panel B, female), heart failure hospitalization (Panel C, male; Panel D, female) across different stimulation strategies and gender after propensity score matching during the 5-year follow-up period. *Analyzed in 1-year follow-up period
